# Advanced magnetic resonance imaging detects altered placental development in pregnancies affected by congenital heart disease

**DOI:** 10.1038/s41598-024-63087-8

**Published:** 2024-05-29

**Authors:** Daniel Cromb, Paddy J. Slator, Megan Hall, Anthony Price, Daniel C. Alexander, Serena J. Counsell, Jana Hutter

**Affiliations:** 1 Centre for the Developing Brain, School of Biomedical Engineering and Imaging Sciences, King’s College London, London, SE1 7EH UK; 2 Centre for Medical Engineering, School of Biomedical Engineering and Imaging Sciences, King’s College London, London, UK; 3 Centre for Medical Image Computing, Department of Computer Science, University College London, London, UK; 4 School of Computer Science and Informatics, Cardiff University, Cardiff, UK; 5 Cardiff University Brain Research Imaging Centre, School of Psychology, Cardiff University, Cardiff, UK; 6 Smart Imaging Lab, Radiological Institute, University Hospital Erlangen, Erlangen, Germany

**Keywords:** Paediatric research, Translational research, Magnetic resonance imaging, Congenital heart defects, Congenital heart defects, Magnetic resonance imaging, Diagnostic markers, Outcomes research

## Abstract

Congenital heart disease (CHD) is the most common congenital malformation and is associated with adverse neurodevelopmental outcomes. The placenta is crucial for healthy fetal development and placental development is altered in pregnancy when the fetus has CHD. This study utilized advanced combined diffusion-relaxation MRI and a data-driven analysis technique to test the hypothesis that placental microstructure and perfusion are altered in CHD-affected pregnancies. 48 participants (36 controls, 12 CHD) underwent 67 MRI scans (50 control, 17 CHD). Significant differences in the weighting of two independent placental and uterine-wall tissue components were identified between the CHD and control groups (both p_FDR_ < 0.001), with changes most evident after 30 weeks gestation. A significant trend over gestation in weighting for a third independent tissue component was also observed in the CHD cohort (R = 0.50, p_FDR_ = 0.04), but not in controls. These findings add to existing evidence that placental development is altered in CHD. The results may reflect alterations in placental perfusion or the changes in fetal-placental flow, villous structure and maturation that occur in CHD. Further research is needed to validate and better understand these findings and to understand the relationship between placental development, CHD, and its neurodevelopmental implications.

## Introduction

The placenta delivers oxygen and nutrients to the developing fetus, removes carbon dioxide and waste products, and performs a host of endocrine and immune functions. Placental development and function are key to healthy fetal development and impaired development can be linked to future health outcomes^1–3^.

Congenital heart disease (CHD) is the most common congenital malformation, affecting ~ 1% of live-births^4^, and is associated with impaired brain development and adverse neurodevelopmental outcomes^5^ which persist into adulthood^6–8^. Previous studies suggest that placental development is altered in pregnancies where the fetus has CHD: placental weight and volume can differ, depending on the type of CHD^9,10^ and gross morphological differences, such as the umbilical cord insertion site, have been recorded^11,12^. Histological studies have identified differences^13,14^, including an increased incidence of thrombosis and infarction^15^ and vascular malperfusion lesions^16,17^. In certain CHD subtypes, placental gene expression and nutrient transfer is abnormal^18^ and MR imaging studies, although limited in number, have revealed differences in placental function^19,20^.

The fetal heart and placenta are both embryological fetal vascular organs, with shared expressed gene pathways^21,22^. It is plausible that placental vasculature may also be disrupted in fetal CHD, although the mechanisms remain unclear^23^. Recently, focus has shifted to the “heart-brain-placenta axis”^24–28^ to improve our understanding of the impaired brain and placental development seen in CHD. For example, there was a trend towards more severe brain injury in neonates with CHD when placental pathology was present^13^ and individuals with both CHD *and* placental abnormalities have significantly lower cognitive and motor performance scores in early childhood^29^, although understanding the exact relationship between these factors is complex.

Identifying differences in structure and function of the CHD placenta is now a key area for research in the fetal CHD population^23,30^. Placental histopathological examination is undoubtedly helpful for identifying gross morphological and microscopic changes in the CHD placenta^31^. However, placental histopathology has been likened to performing an autopsy, where important information only becomes available after delivery, and by which time placental structure and functional properties have changed substantially^32–34^. There is therefore a need for techniques to quantify placental health, structure and function in-utero.

Ultrasound, the current 'gold-standard' screening tool during pregnancy, is not suitable for assessing placental function or microstructure. The complex cascade of events, from inflow of highly oxygenated maternal blood through the spiral arteries, to exchange across the syncytiotrophoblast calls for comprehensive in-utero investigations exploring factors such as perfusion and tissue oxygenation. Placental MRI is a safe, non-invasive technique allowing such in-utero analysis, with both T2*-relaxation and diffusion imaging techniques used to assess placental function and microstructure throughout gestation^35–39^.

T2*-relaxation exploits the blood oxygen level dependent effect linking shorter T2* values to, amongst other factors, a higher concentration of deoxygenated hemoglobin, and is often interpreted as a proxy for placental oxygenation or function^40,41^. Since placental T2* is sensitive to the balance between oxygenated maternal blood delivery and fetal oxygen demand, it is particularly well-suited to identify altered placental oxygenation or blood flow patterns, as might be seen in CHD^20,39,41–45^. Diffusion MRI is sensitive to the speed and directionality of motion of water molecules and thereby provides information relating to tissue microstructure. Importantly, the combination of an acquisition comprising multiple MR images and a mathematical model unlocks sensitivity to structures much smaller than the voxel size^46^.

Emerging combined diffusion-relaxation MRI techniques enable the acquisition of multi-modal diffusion and relaxation data in a single, efficient MR scan, instead of the conventional sequential scans. Measuring diffusion and relaxation simultaneously allows disentanglement of distinct tissue microenvironments that cannot be distinguished with T2*-relaxation or diffusion MRI alone^47,48^.

**Figure 1:**
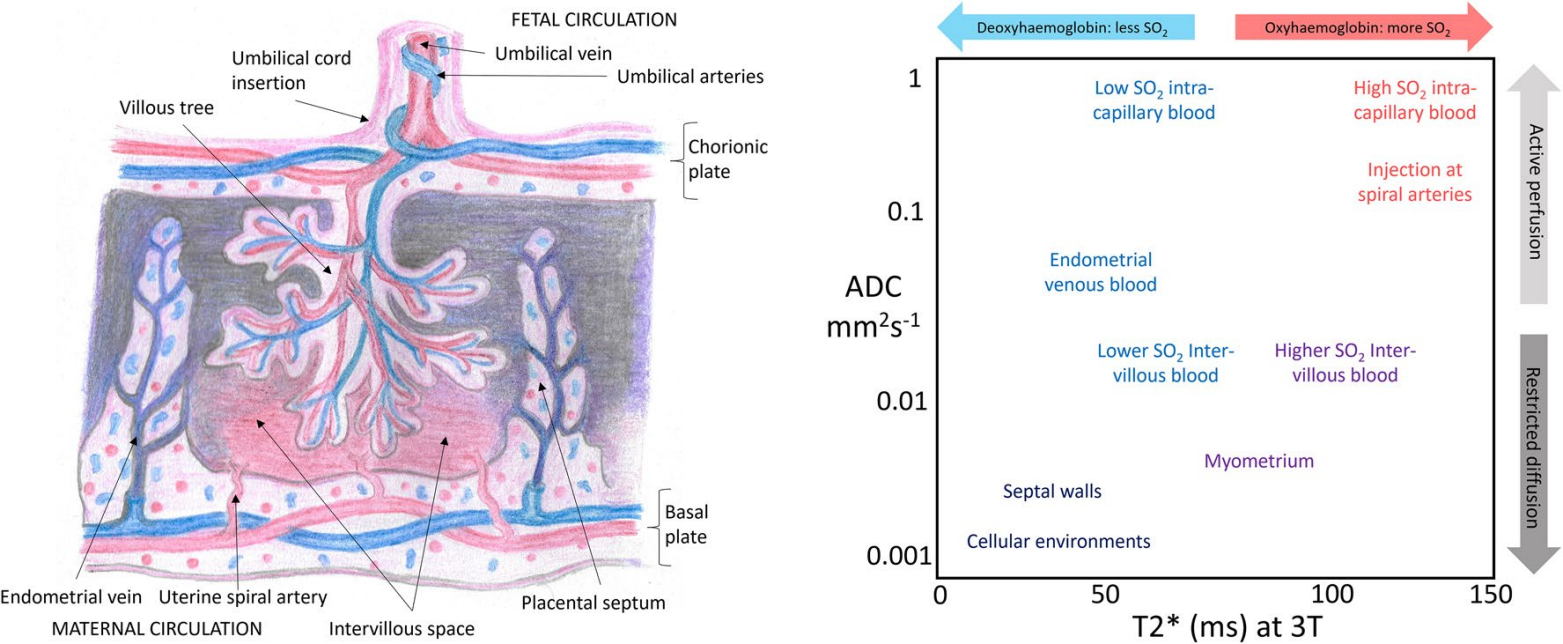
Placental schematic depicting placental anatomy and structures at ~ 32 weeks gestation (left), and the corresponding speculative tissue environments characterized by their T2*-ADC properties (right).

Combined diffusion-relaxation MRI has been demonstrated in the placenta using the ZEBRA^49^ technique and shows promise for identifying placental dysfunction^48^. Additionally, an unsupervised, data-driven analysis technique known as *InSpect* has been developed, enabling simultaneous assessment of placental oxygenation, microstructure and microcirculation from T2*-diffusion data^48,50^. Here, the MRI signal is modelled as the sum of signals arising from different tissue and blood compartments. The decomposition of the signal into components can then be learned in an unsupervised manner from control population data and applied to placental MRI data acquired in cases where placental development may be altered. Figure 1 demonstrates how combined T2* and Apparent Diffusion Coefficient (ADC) values at 3 T could be interpreted as representing different placental tissues using this technique.

This study utilized advanced combined diffusion-relaxation MRI and an unsupervised, data-driven analysis technique (InSpect) to test the hypothesis that placental microstructure and perfusion are altered in-utero in pregnancies affected by CHD.

## Results

### Participant demographics

48 individual participants (36 controls, 12 CHD) satisfied the inclusion criteria, resulting in 3 Tesla MR imaging data from 67 scans in total (50 control, 17 CHD). Maternal demographics for all participants are in Table 1. There was a significant difference in maternal age at scan between the control and CHD samples, (median age 35.0 years (33.2–37.1) vs 32.3 years (27.9–34.9) respectively, p = 0.008). There was no difference in scan GA or BMI between groups. Diagnoses included in the CHD cohort were: Coarctation of the aorta (CoA) = 4; Tetralogy of Fallot (ToF) = 1; Transposition of the Great Arteries (TGA) = 3; Hypoplastic Left Heart Syndrome (HLHS) = 3; Truncus Arteriosus (TA) = 1.

**Table 1:** Maternal participant demographics.

Cohort	Participants	Scans	GA at scan (weeks)*	P^✝^	Maternal BMI at scan (kg/m^2^)*	P^✝^	Maternal age at scan (years)*	P^✝^
Control	36	50	29.9 (26.9–32.6)	–	26.2 (± 3.0)	–	35.0 (33.2–37.1)	–
CHD	12	17	32.6 (28.7–34.0)	0.09	26.3 (± 4.0)	0.91	32.3 (27.9–34.9)	**0.008**
All	48	67	30.7 (27.4–33.1)	–	26.2 (± 3.0)	–	34.6 (32.5–36.7)	–

*Values given are mean (± standard deviation) for parametric data, or median (25th centile-75th centile) for non-parametric data.

^✝^P-value for comparison between mean or median values for control and CHD cohort and control and PIH cohort separately. Results in bold are considered significant.

### Placental volume, T2* and ADC measurements

**Figure 2:**
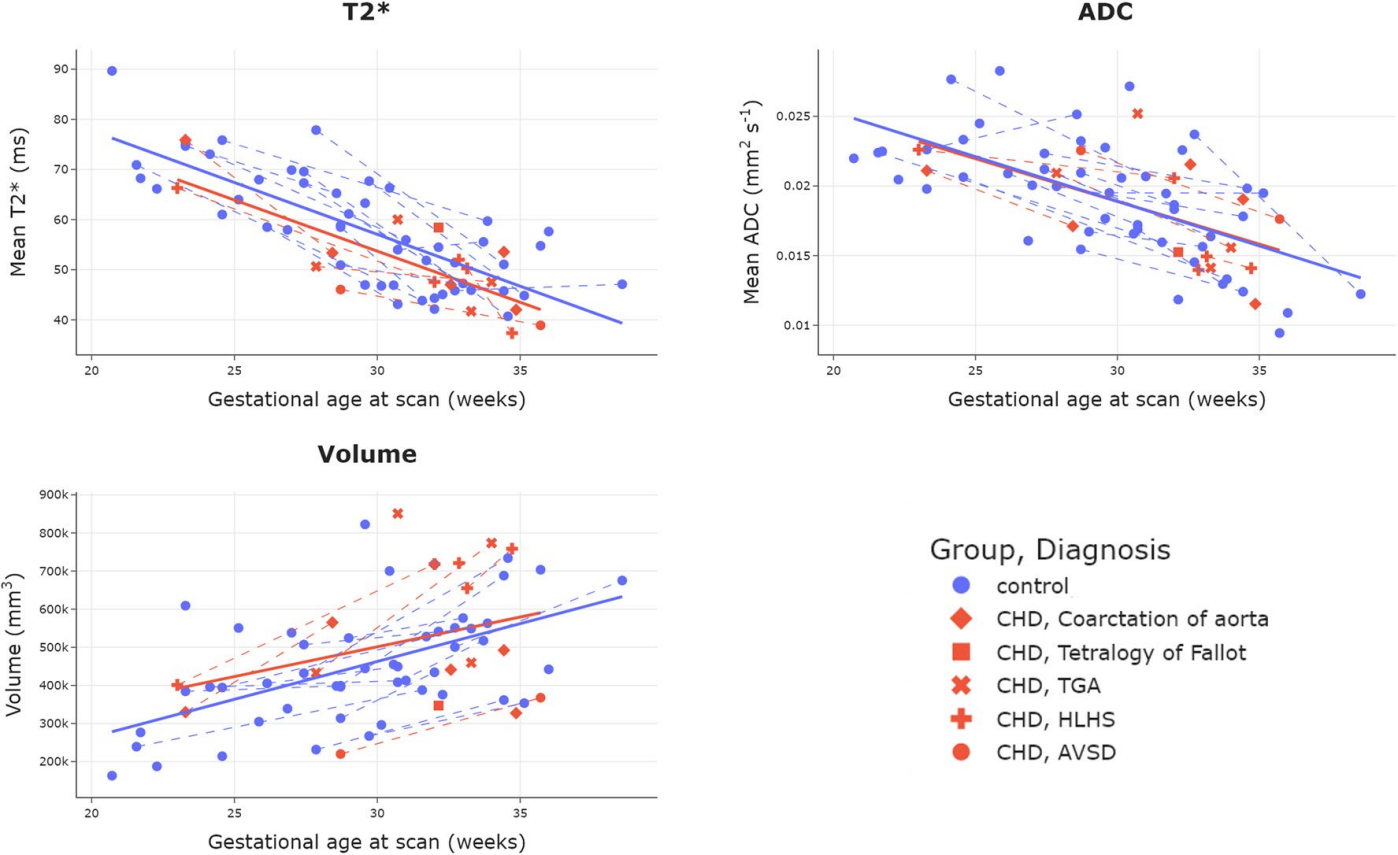
Plots showing mean placental and adjacent uterine wall T2* (top left), ADC (top right) and volume (bottom left) over gestation for all scans. Dotted lines join participants who had two scans.

Results of ROI volume, mean T2* and mean ADC values are shown in Table 2. After accounting for scan GA and maternal age, mean T2* was significantly lower in the CHD sample (mean T2*: CHD 51.1 ± 9.9 ms, Control 58.1 ± 11.4 ms, p = 0.012), but there were no significant differences in volume or mean ADC values between groups. Plots showing mean placental volume, T2* and ADC values across gestation for all 67 scans are shown in Fig. 2. Placental volume increased significantly with GA (R = 0.50, p < 0.0001). Both mean T2* and ADC decreased significantly with GA (R = − 0.78, p < 0.0001; R = -0.63, p < 0.0001 respectively).

**Table 2:** MRI derived placental characteristics.

Cohort	Placental volume (mm^3^)*	P^✝^	Mean placental T2* (ms)	P^**✝**^	Mean placental ADC	P^**✝**^
Control	453,000 (± 152,000)	–	58 (47–67)	–	0.020 (0.016–0.022)	–
CHD	512,000 (± 190,000)	0.37	51 (46–54)	**0.12**	0.017 (0.014–0.021)	0.97
All	471,000 (± 163,000)	–	54 (47–65)	–	0.019 (0.016–0.022)	–

*Values given are mean (± standard deviation) for parametric data, or median (25th centile-75th centile) for non-parametric data.

^✝^P-value from ANCOVA between control and CHD cohort, after accounting for gestational age at scan and maternal age. Results in bold are considered significant.

### Interpreting microenvironments

**Figure 3:**
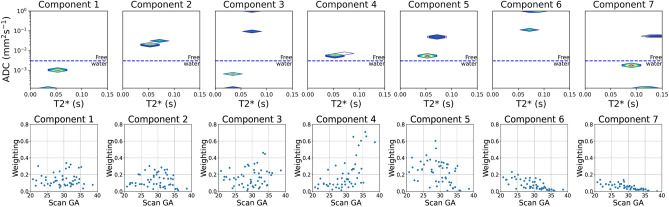
Seven component InSpect fit to the whole placenta and uterine wall ROI data from 36 control participants, assuming the T2*‐ADC model described in Eq. (1). Top-row: T2*-ADC spectra for each component, which are shared across all 36 participants. Each T2*-ADC spectra represents the T2*-ADC distribution for that component, with each spectral peak reflecting a population with distinct T2* and ADC, and hence microstructural and microcirculatory, properties. Bottom row: plots showing the mean component weightings for each participant across gestation.

The derived T2*-ADC control spectra for each of the seven InSpect components, after running ‘full InSpect’ on data from 36 control participants, are shown in Fig. 3. Each T2*-ADC spectra represents the T2*-ADC distribution for that component, with each spectral peak reflecting a population with distinct T2* and ADC, and hence microstructural and microcirculatory, properties. Plots showing the mean MRI signal weighting for each ROI as a proportion of the total signal for each of the seven components across gestation, for all control participants, are also shown.

**Figure 4:**
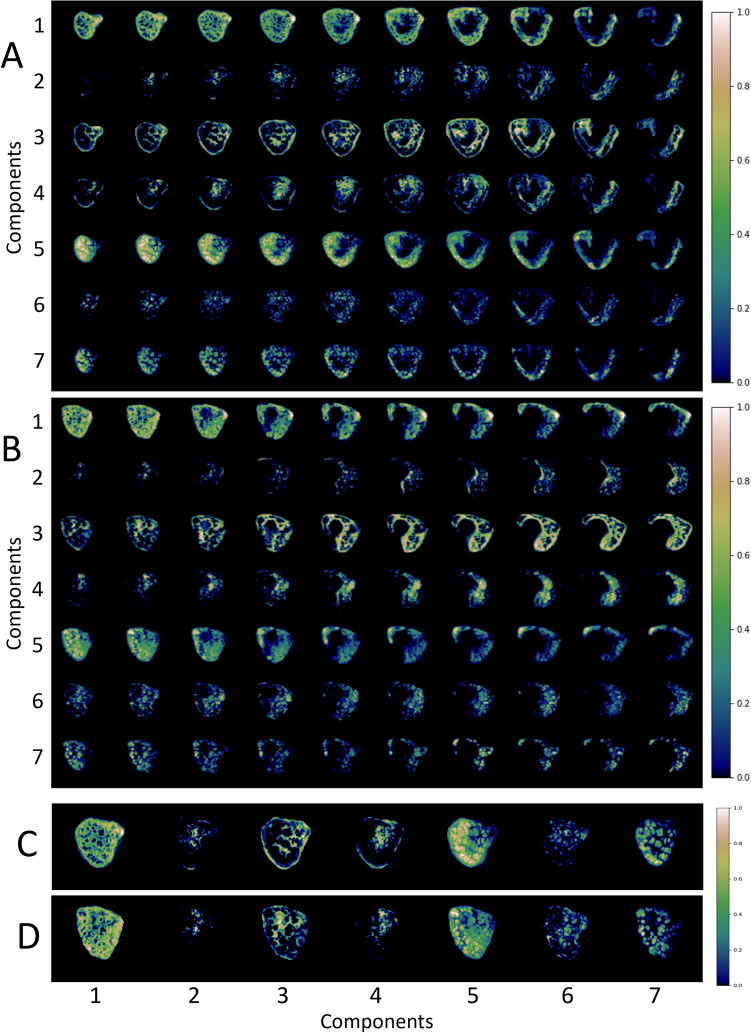
Placental composite images for two participants acquired at comparable gestational ages, showing the middle ten slices. Panel (**A**) is from a control participant, acquired at 34^+3^weeks. Panel (**B**) is from a CHD participant, acquired at 34^+5^weeks. The rows in panel A and B represent each component (1–7) and the columns represent slices through the ROI (left-to-right = anterior-to-posterior). Panel (**C**) and panel (**D**) show selected mid-placental slices from the same control (**C**) and CHD (**D**) dataset, highlighting the spatial location of each component at a gestational age of 34–35 weeks, with the columns representing components 1–7. The color scale (0–1) is the same for all images, representing the proportion of MR signal present in each voxel for each component.

Figure 4 shows composite spatial maps for both a control and a CHD participant, acquired at comparable GAs, highlighting differences in voxelwise weightings for all seven components (rows) and all placental slices (columns). Component one has two spectral peaks, both with relatively low T2* (< 0.06 s) and ADC (< 0.001 mm^2^ s^−1^) values, representing poorly oxygenated tissues with lower diffusivity. The spatial maps for this component show the highest signal in the periphery of placental lobules. Component three contains some spectral peaks with higher T2* (> 0.07 s) and ADC (> 0.1 mm^2^ s^−1^) values and, particularly at later gestations, is conspicuously absent from within placental lobules. It has a high signal towards the edge of the placenta and adjacent uterine wall and could be interpreted as representing connective tissue structures such as placental septa, as well as blood in vasculature within the uterine wall. Component seven has multiple spectral peaks, all with relatively high T2* values (> 0.09 s), reflecting well oxygenated tissues. The spatial maps show it is confined within the placental lobules, with ‘hot-spots’ at the center of each lobule, and may therefore represent blood flowing into the lobules via uterine spiral arteries, before being slowed abruptly as it travels through the villous tree architecture at the fetal-maternal exchange surface.

An overview of the spectral peaks, mean signal-weighting contribution, how these weightings change over gestation and the spatial distribution for each component, used for interpretation of the underlying tissue environments, are in Supplementary Table S1. Selected mid-placental slices for all participants for each component are in Supplementary Figs. S1–S7.

### Component weighting plots

**Figure 5:**
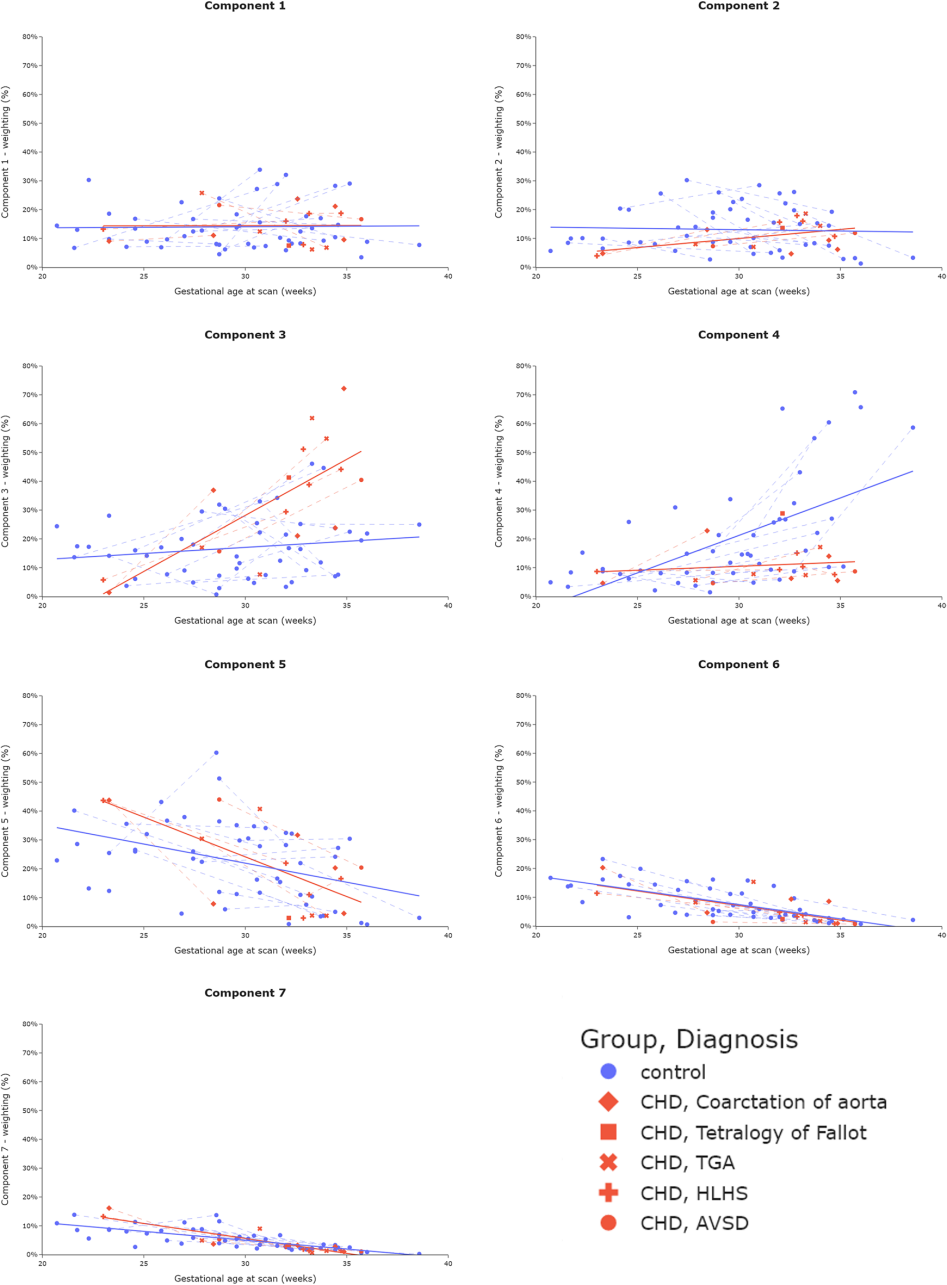
Mean ROI (placenta and adjacent uterine wall) component weightings across gestation from 67 scans (50 control, 17 CHD). Component weighting ranges (y-axis) are kept consistent (0–80%) to aid interpretation as to the overall contribution to the MR signal from each component. Dotted lines join participants who underwent two scans.

All component weighting plots are shown in Fig. 5.

After accounting for GA at scan and maternal age, there was a significant difference in mean ROI weightings between control and CHD groups for component three and component four (both p_FDR_ < 0.001).

For control data, component four was the only component to show a significant increase across gestation, occurring most noticeably after 30 weeks (R = 0.60, p_FDR_ < 0.001). Components five, six and seven show a significant decrease (R = − 0.40, p_FDR_ = 0.004; R = − 0.71, p_FDR_ < 0.001; R = -0.74, p_FDR_ < 0.001 respectively).

For CHD data, components two and three showed a significant increase across gestation (R = 0.50, p_FDR_ = 0.040; R = 0.73, p_FDR_ = 0.0013 respectively), whereas components five, six and seven show a significant decrease (R = − 0.67, p_FDR_ = 0.0033; R = − 0.68, p_FDR_ = 0.0025; R = − 0.89, p_FDR_ < 0.001 respectively).

## Discussion

This is the first study utilizing combined diffusion-relaxation MRI to explore placental structure and function in-utero in CHD-affected pregnancies. We used a data-driven approach simultaneously sensitive to oxygenation, microstructure and microcirculation^50^ to show that independently derived placental and adjacent uterine wall tissue environments change significantly during key periods of fetal development, between 20 and 40 weeks gestation, in both normal pregnancies and those where the fetus has CHD.

For multiple components, different trends over gestation for control and CHD data are observed (Fig. 5). The weighting of component three increases noticeably after 30 weeks in CHD cases, in contrast to the control sample. For component four, the increase in weighting after 30 weeks in control participants is not reflected in the CHD data. Based on their MR properties and spatial distribution (see Supplementary Table S1), component three could represent poorly perfused structures such as placental septa, as well as blood within vasculature in the uterine wall and component four may represent blood returning from the fetus and within maternal uterine veins. Importantly, however, this result is independent of any interpretation of the specific placental microenvironments these components represent. A significant trend over gestation in weighting for component 2 was also observed in the CHD cohort, but not in the control cohort.

These findings add to existing evidence that placental development is altered in CHD, and complements research using combined diffusion-relaxation MRI to identify placental compartments with distinct T2*-ADC combinations^51^ and abnormal placentation associated with pregnancy-related conditions PE or FGR^48,52^. The results reported here have the potential to help with understanding of the interlinked pathways between placental and cardiac development^22^.

One hypothesis is that changes observed here may reflect alterations in placental perfusion seen in CHD^43,53^. However, given the small difference in mean placental T2* between groups, and the difference in trajectories between T2* and the weighting of components three and four over gestation, it is unlikely that reduced perfusion alone is driving these differences. Changes in fetal-placental flow^54^ and villous structure^18^ that occur in CHD may also be contributing. As pregnancy advances, specific microstructural changes occur within the placenta, particularly in the third trimester^55^, including terminal villi development^56^ and fetal villous angiogenesis^57^. This is an important adaptation that ensures efficient oxygen and nutrient exchange in the later stages of gestation, to meet fetal demands, but as terminal villous development is directly influenced by placental oxygen levels in normal pregnancy^58^, this process may be altered in CHD. Altered villous maturation, consistent with an ‘immature’ placental microvasculature, could also be preventing maximal oxygenation of fetal blood in CHD^23^. It is interesting to note that the GA after which the differences in the weighting of components three and four between groups becomes most apparent—30 weeks—is consistent with the GA at which volumetric brain development also deviates from normal in fetuses with CHD^59^.

The approach we have used involves no a-priori understanding of different placental compartments or microstructural environments, but identifies them based on shared T2*-ADC characteristics and an understanding of placental structure (Fig. 1). The fetal circulation is intra-capillary and has a relatively low oxygen saturation at the exchange surface, whereas the maternal circulation enters the placenta as a highly-saturated blood pool, but is extravascular in the human placenta^47^. Despite this complex, heterogeneous structure, previous studies have used mean whole-placental MRI biomarkers^60,61^, making interpretation of the results challenging. Identifying unique placental tissue compartments that might be altered in CHD fits the inherent complexity of the placenta and thus helps provide a focus for future research studies. The associated spatial maps aid in localisation of these compartments, helping differentiate tissue environments as they change throughout the placenta, i.e. from basal to decidual plate.

Additionally, the complexity of placental physiology benefits from a comprehensive assessment approach, such as this combined use of diffusion-relaxation and a bespoke analysis tool like InSpect. Whilst a small but significant decrease in whole placental T2* was identified in the CHD cohort, there was no significant difference in whole placental ADC values between groups. This further highlights the enhanced sensitivity of InSpect to detect changes in placental function and microstructure beyond the use of T2* or ADC independently.

However, it is important to emphasize that using a data-driven approach means components don’t necessarily demonstrate one-to-one mapping of placental compartments such as “fetal" and “maternal", or “intracellular" and “extracellular", but can also reflect combined tissue environments. Placental anatomy means tissue environments with different T2*-ADC properties can sit in close proximity, i.e. at the villous exchange surface, where pooled maternal blood lies close to fast flowing fetal blood in small diameter vessels, or at the umbilical cord insertion site, where similar sized vessels containing blood with very different T2* properties intertwine.

It is also worth highlighting that InSpect provides the *proportion* or *weighting* of the MR signal in each voxel that each component represents. This results in component weightings that are intrinsically linked and may explain why there appears to be such a ‘reciprocal’ change in components 3 and 4, since a reduction in the weighting for one component necessitates an increase in another. However, this result is independent of any interpretation of the specific placental microenvironments and suggests a clear difference in at least one compartment. Furthermore, any changes in component weighting, i.e. those seen over gestation, or between groups, suggests that the tissue environment(s) represented by that component change *as a proportion of total placental volume* over gestation, and not necessarily that there is a change in *absolute* volume.

As expected, we show that placental volume increases with advancing gestation. Consistent with previous work, we also identify a decrease in both T2* and ADC values with advancing gestations^39,40,62^, which appear consistent in healthy participants undergoing two scans^63^.

This study is limited by relatively small numbers at early gestations. The CHD cohort is also diagnostically heterogenous, and different diagnoses may impact placental development or function in different ways^13,15^. However, all CHD diagnoses were critical or serious, which is where the greatest alterations in placental development in CHD might be expected^12,64^. Different types of CHD may affect fetal-placental flows in different ways^43,65,66^, or involve different genes linking placental and vascular development^18^, so future work with larger cohorts is needed to explore how certain CHD diagnoses or physiologies might be associated with impaired placental development.

Using a data-driven technique such as InSpect involves speculation as to the underlying tissue environments or microstructures represented. There is currently no ‘ground truth’. We also did not collect ultrasound information, such as that relating to uterine artery resistance or dopplers, which may be helpful for establishing the presence of uteroplacental dysfunction^43^. Future work should involve attempts to validate and better understand these findings, either through histopathological examination, by invasive sampling^67^ or in comparison with complementary ultrasound techniques^68^.

Future work including data from other cohorts where placental dysfunction is better understood/characterised, for comparison to both CHD and control placentas, would also be beneficial. For example, others have previously identified associations between fetal CHD and maternal hypertensive disorders of pregnancy^69,70^, including the risk of pre-eclampsia^16^, hinting at a potential common aetiology, which could be explored using this approach in future.

Furthermore, we did not collect data related to maternal or fetal haematinics. However, it is plausible that levels of fetal or maternal haemoglobin influence placental T2* values. Given that maternal haemoglobin levels change over gestation^71^, and that fetal haematinics are affected by both impaired placentation and CHD^72,73^, future work should attempt to capture and include this information.

The trends over GA of several component weightings, seen in both CHD and control placentas, could reflect normal changes in the microstructure the placenta as pregnancy advances^31,32,74^, with the corresponding differences in tissue microstructure and perfusion that occur^75^, and in future could serve as imaging biomarkers of both normal and abnormal placental development. Given enough data from typically developing placentas, InSpect could also be used to generate a quantitative ‘placental abnormality’ score, taking into account the deviation from normal of each component weighting for a given GA. This would enable both quantifiable analysis of impaired placentation and help identify where within the placenta this occurs.

## Conclusions

We report using combined diffusion-relaxation MRI and a data-driven approach to detect altered placental tissue environments in pregnancies affected by fetal CHD, with changes most evident after 30 weeks gestation. We speculate that these changes are driven by impaired perfusion and microstructure in the CHD placenta, although future work is needed to definitively link these imaging findings to potential alterations in the underlying placental structure and function.

## Materials and methods

### Ethics and recruitment

Data were acquired as part of The Congenital Heart Disease Imaging Programme (CHIP) at St. Thomas’ Hospital in London. All methods were carried out in accordance with relevant guidelines and regulations and all experimental protocols were approved by the NHS Wales REC4 Research Ethics Committee [NHS REC 21/WA/0075]. Control participants experiencing a low-risk pregnancy, with the absence of pregnancy-induced hypertension (PIH), preeclampsia (PE), fetal growth restriction (FGR), or gestational diabetes (GD) at the time of enrolment, were recruited after their antenatal booking or screening appointments. Participants with a fetus with severe or critical CHD, as defined previously^76^, confirmed on fetal echocardiography, were recruited from the fetal cardiology clinic. Participants with PIH, PE, FGR, GD, or where the fetus had confirmed genetic abnormalities were also excluded from the CHD cohort. All participants were invited to have up to two fetal MRI scans from a gestational age of 20 weeks.

Data were subsequently excluded if the pregnancy resulted in a delivery before 37 weeks gestational age (GA), if PIH, PE, FGR or GD were newly diagnosed between scan and delivery, if any genetic abnormalities were detected on antenatal testing, or if any significant incidental fetal or placental findings were reported on imaging. Data sets with insufficient quality, including cropping of the placenta, extensive geometric distortion artifacts, or visible contractions during the scan were also excluded.

### Image acquisition and reconstruction

Informed, written consent was obtained from all subjects prior to imaging. Images were acquired on a Philips Achieva 3 T scanner using a 32-channel surface coil. All imaging was performed in supine position with frequent verbal interaction, continuous heart rate and oxygen saturation monitoring, and blood-pressure measurements at 10 min intervals.

Following a pilot scan and B0 and B1 calibration scans, anatomical imaging using T2-weighted turbo-spin-echo sequences, as well as a multi-echo gradient-echo sequence, a combined T2*-diffusion scan (ZEBRA^49^) was performed, with parameters defined in Table 3. Acquisition time for this sequence was 8 m 30 s.

**Table 3:** Combined T2*-ADC multi-echo gradient-echo MRI scan acquisition parameters.

Orientation: Coronal plane to maternal habitus, FOV = 300 × 320 × 84 mm
Resolution: 3 mm^3^ isotropic
Echo Time = (78, 114, 150, 186) ms, Repetition Time = 7.5 ms, SENSE factor = 2.5
b = (5, 10, 25, 50, 100, 200, 400, 600, 1200, 1600) s mm^−2^; 3 directions
b = 18 s mm^−2^; 8 directions
b = 36 s mm^−2^; 7 directions
b = 800 s mm^−2^; 15 directions

The acquired data were then anonymised and reconstructed using in-house tools, including denoising and motion-correction, as previously described^49^. The reproducibility of this T2*-diffusion sequence has been demonstrated in MR phantom, adult brain, and placental studies^49,63^.

### T2* and ADC mapping

**Figure 6:**
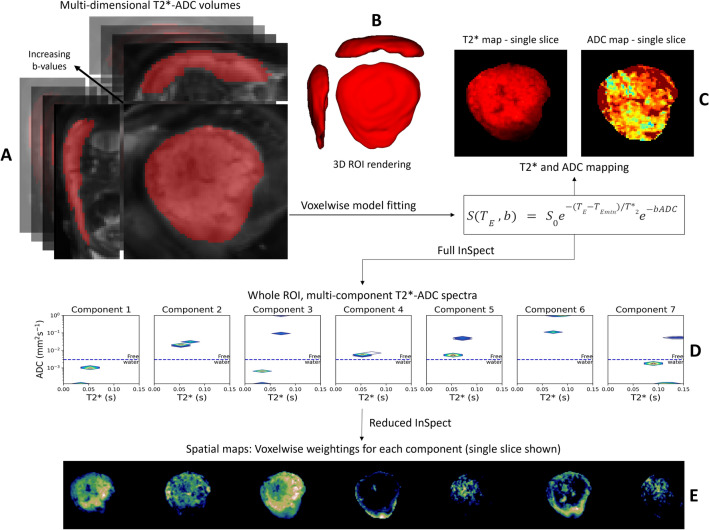
Data flow. Manual regions of interest (ROI) (placental and adjacent uterine wall mask) overlaid on the first multi-dimensional volume (b = 0, TE = 78 ms) of the acquired diffusion-relaxation data (**A**), with the corresponding 3D-ROI rendering adjacent (**B**). After fitting Eq. (1) voxelwise to all voxels in this 3D-ROI, T2* and ADC maps were generated. These are shown for a selected coronal slice from a healthy placenta, acquired at 28^+4^weeks gestation (**C**). Subsequently, ‘full’ InSpect was run on data from 36 control participants, to generate the T2*-ADC spectra associated with each of the seven components (**D**), and also calculate the voxelwise weightings of each component, shown here for the same selected coronal slice (**E**). These spectra were then used to infer voxelwise spatial maps for all CHD scans and additional ‘repeat’ scans from the control sample (‘reduced InSpect’).

Figure 6 outlines the data-processing workflow. First, a region of interest (ROI) containing the whole placenta and adjacent uterine wall section was manually segmented on the anonymised first b = 0 image with the lowest echo time, by an experienced clinician, who was blinded to the maternal demographics and fetal diagnosis. The T2*-ADC model described in Eq. (1) was then fit voxelwise using a modified version of the diffusion microstructure imaging in python toolbox^77^, as described in^63^, enabling calculation of the mean T2* and ADC values for the whole ROI.

### Data-driven analysis with InSpect

We ran InSpect on the first scans from all 36 control participants using the InSpect toolbox (https://github.com/PaddySlator/inspect) (Fig. 6). Seven InSpect components were chosen as this number has previously been shown to best explain the placental and adjacent uterine-wall T2*-diffusion signal^50^. This full InSpect is a process akin to independent component analysis, albeit under the assumption that the data is generated by the underlying dynamics of Eq. (1). Full InSpect hence identified seven components in the data, with each component having a corresponding T2*-ADC spectra (e.g. Fig. 6). The spectral peaks in these T2*-ADC spectra represent different tissue microenvironments within the ROI. InSpect has no a-priori information about the tissue or organ being imaged. For placental imaging, this full InSpect analysis is equivalent to solving an inverse problem, where the algorithm estimates the proportions of each tissue component that would need to be present in an image voxel to yield the observed T2* and ADC measurements. The relative weighting of each of these components is calculated voxelwise during this full InSpect process, allowing maps quantifying the spatial distribution and relative amount of each component present in every voxel to be created (e.g. Fig. 6). These components and their corresponding T2*-ADC spectral peaks in data from control participants were assumed to be representative of typically developing placentas. We then quantified how the relative fractions of these components change over GA.

1 $$S({T}_{E} ,b) = {S}_{0}{e}^{-{(T}_{E}-{T}_{Emin})/{T}_{2}^{*}}{e}^{-bADC}$$

where S_0_ is the signal at the proton density (b = 0), T_E_ is the echo time, T_E_ is the shortest echo time acquired, ${T}_{2}^{*}$ is the effective transverse-relaxation time, *b* is the b-value and ADC is the apparent diffusion coefficient.

These spectra were then used to infer voxelwise spatial maps for all CHD scans and additional ‘repeat’ control scans, in a process we term ‘reduced InSpect’. Reduced InSpect fixes the values of the T2*-ADC spectra associated with each component, only calculating the corresponding maps, ensuring the components are identical for all data being analyzed with the additional benefit of computational efficiency. The initial phase, termed full InSpect, involves learning the components that best represent the data, while the subsequent step, reduced InSpect, essentially projects the new data onto these learned components. The results of these analyses were used to determine how much each component differs from normal for each CHD dataset.

### Interpreting microenvironments and plotting component weightings

Next, the overall contribution, or weighting, of each component of the InSpect analysis was plotted against GA for each dataset. The MR tissue properties of each component were then assessed, taking into account their T2*-ADC spectral peaks, with relatively higher T2* values representing more well-oxygenated tissue and higher ADC values (above free-water = 0.3 mm^2^ s^−1^) representing perfusing or fast-flowing blood, usually interpreted as within vasculature. Combining this information with the MR signal weighting contributed by each component, how the weightings change over gestation, and how the components are spatially distributed, enabled speculations about the tissue microenvironments encoded by each component to be made.

### Statistical analyses

A Shapiro–Wilk test was used to test normality. The Mann–Whitney U-test was used to compare non-normally distributed values of GA at scan and maternal age at scan between groups. A T-test was used to compare the normally distributed values of maternal BMI between groups. An ANCOVA was used to compare placental volume, T2*, ADC between groups, after accounting for GA at scan and maternal age. Pearson's correlation coefficient was calculated to determine the direction and strength of the relationship between placental volume, T2* and ADC across the GA ranges studied. For these analyses, uncorrected p-values (reported as p) less than 0.05 were considered significant.

An ANCOVA was used to compare mean component weightings between groups, after accounting for GA at scan and maternal age, for each of the seven components. Pearson's correlation coefficient was calculated to determine the direction and strength of the relationship between component weightings across the GA ranges studied, for each of the seven components. For each of these analyses involving component weightings, Benjamini and Hochberg false discovery rate (FDR) correction was applied to correct for multiple comparisons (reported as P_FDR_), as seven independent components were being analysed. Here, corrected p-values (reported as p_FDR_) less than 0.05 were considered significant. All statistical analyses were performed using statsmodels v0.13.2^78^ and Jupyter Notebook, python3.

### Supplementary Information

Supplementary Information.

## Data Availability

The datasets analysed during the current study are available from the corresponding author on reasonable request.
