# Supplementary material for: Advanced magnetic resonance imaging detects altered placental development in pregnancies affected by congenital heart disease
Source: Sci Rep. 2024 May 29;14:12357. doi: 10.1038/s41598-024-63087-8 (PMC11136986; doi:10.1038/s41598-024-63087-8)
Supplement: Supplementary file 1 — Supplementary Information. [file 41598_2024_63087_MOESM1_ESM.pdf]

Supplementary material

<u>Supplemental-Figure 1</u>: Component mosaics, composed of selected ‘mid-placental’ slices for all control placentas (left panel) and all CHD placentas (right panel). In all panels, gestational age at scan is earliest (20 weeks) in the top left and latest (36 weeks) in the bottom right, enabling a comparison of component weighting at similar gestational ages. The color scale (0 to 1) is the same for all images, representing the proportion of MR signal present in each voxel for each component.

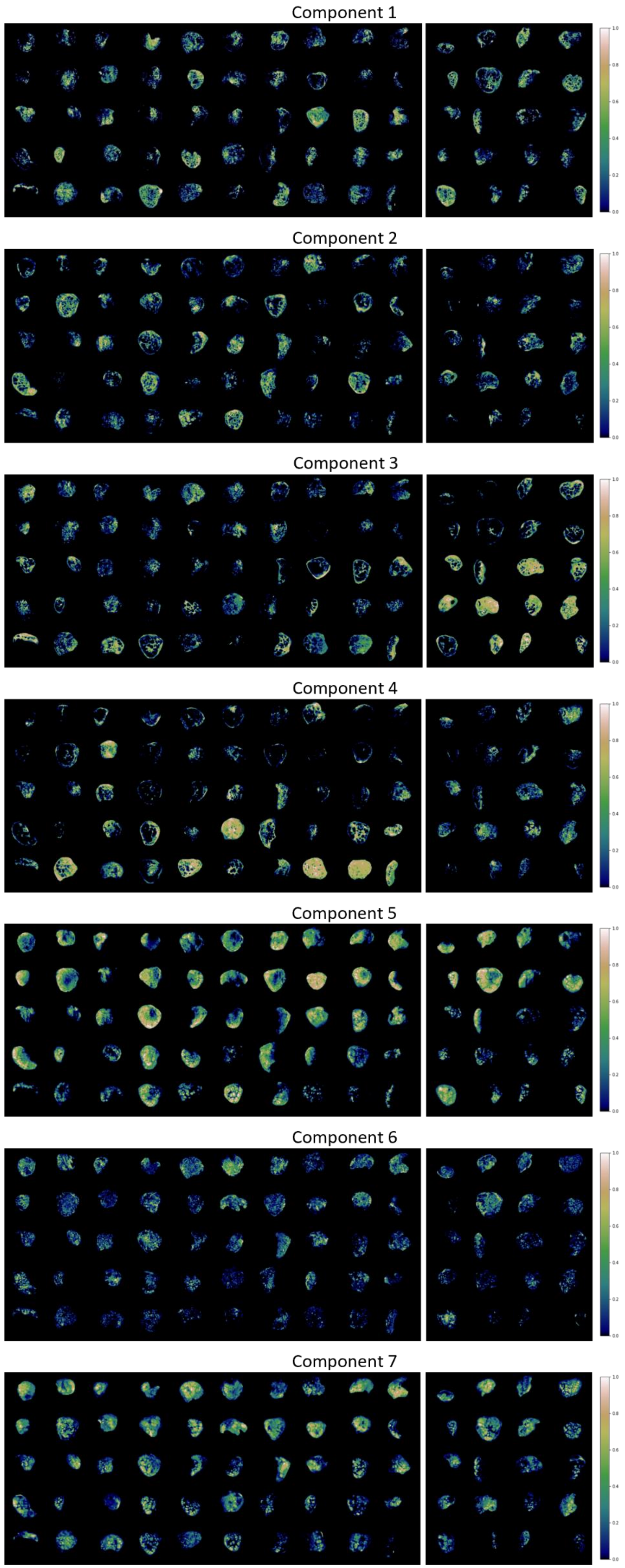
Component 1
Component 2
Component 3
Component 4
Component 5
Component 6
Component 7

<u>Supplementary-Table 1:</u> A summary of the relative T2* and ADC values of spectral peaks for each component, the mean signal weighting contributed by each component over gestation, how this weighting changes over gestation (where applicable) and the speculative interpretation of the underlying tissue environments represented by each component.

Component	Relative T2* value(s)	Relative ADC value(s)	Mean signal contribution	Trend over gestation?	Speculated tissue environments
**1**	Low (<60ms)	Low (0.001-0.01)	14%	NA	Both peaks have relatively low T2* and ADC values. The signal is diffuse at earlier gestations and located more towards the edges of placental lobules later on, suggestive of a combination of slowly percolating blood within the intervillous space and dense connective tissue, i.e. septa within the placenta. A thin 'ring' is sometimes visible around the outer edge of the placenta and may correspond to the surrounding membranes.
**2**	Average (40-80ms)	High (0.03-0.05)	13%	NA	Two closely located peaks, with high ADC values, suggesting water within vasculature. Signal is found more towards the edge of placental lobules, avoiding the center in most cases. There appears to be some uterine wall

Component	Relative T2* value(s)	Relative ADC value(s)	Mean signal contribution	Trend over gestation?	Speculated tissue environments
					and basal plate involvement. It may represent freely perfusing blood traveling in maternal veins draining out of the placental parenchyma.
**3**	Mixed (<40ms and >60ms)	Mixed (<0.001 and >0.1)	17%	NA	Multiple peaks, two with similar T2* values and ADC values below free water, and two with higher T2* ADC values. Spatial maps suggest it may represent blood within and flowing through the uterine wall, as well as connective tissue structures. Its signal is fairly diffuse at earlier gestations, and appears more noticeable in the basal plate and placental septa later on.
**4**	Average (~50ms)	Average (<0.01, but above free water)	20%	Significant increase	There is a peak with an ADC value above that of free water and a T2* value consistent with venous blood. It is prominent towards the edge of the placenta early on and has an obvious change in both overall weighting and spatial location after about 30 weeks gestation, becoming more

Component	Relative T2* value(s)	Relative ADC value(s)	Mean signal contribution	Trend over gestation?	Speculated tissue environments
					noticeable in septa and at the edges of the placental lobules. It may represent blood returning from the fetus and within maternal uterine veins, as well as tissue containing water that is somewhat constrained to the septa, reflecting an increase in fibrous structures in the spaces between placental lobules. The increase in signal weighting seen after ~30 weeks may also be related to an increase in fibrin deposition that occurs around this time, or the changes in flows that result from the changing pressure and pulsatility of maternal blood that takes place across gestation.
**5**	Average (50-70ms)	High (0.05-0.08)	23%	Significant decrease	Two peaks, both with ADC values above free water, similar to that seen in component 2. However, the spatial maps clearly show high signal within the center of placental lobules for this component, particularly at later gestations. The signal 'fades away' towards the periphery of the

Component	Relative T2* value(s)	Relative ADC value(s)	Mean signal contribution	Trend over gestation?	Speculated tissue environments
					lobule and the spectral peaks may correspond to blood within the fetal vasculature and the endometrium.
**6**	High (>70ms)	Very high (0.1-1.0)	8%	Significant decrease	Two peaks, both with high T2* and very high ADC values. It has a diffuse appearance in the spatial maps, although it is absent from the uterine wall, and contributes a relatively low proportion of the total signal overall, with minimal contribution in later gestation. It may reflect well oxygenated blood within the fetal circulation, before the tertiary villi fully develop.
**7**	High (>80ms)	Mixed (<0.001, ~0.001 and ~0.08)	5%	Significant decrease	The peak with high T2* and ADC values may represent jets of fast-flowing maternal blood injected into intervillous space from uterine spiral arteries, contributing to a relatively low overall volume of the placenta, but seen as 'hot-spots' on the spatial maps. The peaks with lower T2* and ADC values may represent blood that has had some oxygen extracted as it

Component	Relative T2* value(s)	Relative ADC value(s)	Mean signal contribution	Trend over gestation?	Speculated tissue environments
					passes the fetal-maternal exchange surface, as well as where diffusion is reduced in the complex architecture of the villous trees.
